# Supplementary material for: Deciphering clinical abbreviations with a privacy protecting machine learning system
Source: Nat Commun. 2022 Dec 2;13:7456. doi: 10.1038/s41467-022-35007-9 (PMC9718734; doi:10.1038/s41467-022-35007-9)
Supplement: Supplementary file 1 — Supplementary Information [file 41467_2022_35007_MOESM1_ESM.pdf]

Supplementary Materials for "Deciphering clinical abbreviations with a privacy protecting machine learning system."

Alvin Rajkomar[1*†],Eric Loreaux[1*], Yuchen Liu[1], Jonas Kemp[1], Benny Li[1], Ming-Jun Chen[1], Yi Zhang[1], Afroz Mohiuddin[1], Juraj Gottweis[1]

[*] These authors contributed equally to this work
[1] Google, Mountain View, California, USA
[†] email: alvinrajkomar@google.com

Supplementary Figure 1: Distribution of abbreviations with naive reverse substitution vs web-scale reverse substitution **2**

Supplementary Figure 2: Number of labeled expansions per snippet in finetuning vs synthetic snippets **3**

Supplementary Figure 3: Diagram of standard, iterative, and elicitive inference techniques **4**

Supplementary Table 1: Results on the synthetic dataset for T5 models trained on MC-WSRS + baseline most common expansion **5**

Supplementary Table 2: Results on all clinical notes datasets for T5 80B model (elicitive inference) fine-tuned on MC-WSRS **6**

Supplementary Table 3: Illustrative human mistakes from human evaluation **7**

Supplementary Table 4: Results on the synthetic dataset for T5 models trained on C4-WSRS **9**

Supplementary Table 5: Results on all clinical notes datasets for T5 11B model (elicitive inference) fine-tuned on C4-WSRS **10**

Supplementary Figure 4: Number of additional inference rounds on synthetic dataset for iterative and elicitive inference **11**

Supplementary Figure 5: General methodology overview **12**

Supplementary Figure 6: Expansion counts in Finetuning data **13**

Supplementary Algorithm 1: Pseudocode for Web-Scale Reverse Substitution **14**

Supplementary Table 6: Miscellaneous examples **16**

Supplementary Figure 1: Distribution of abbreviations with naive reverse substitution vs web-scale reverse substitution

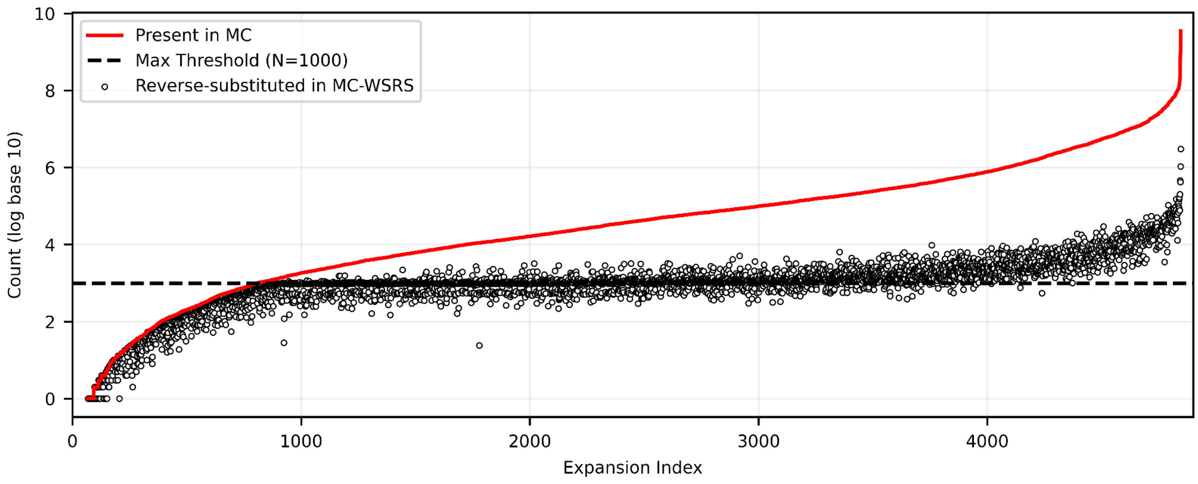

We ranked each expansion in our dictionary by their frequency in the MC web crawl, creating an expansion index from the rank (with the most common long form having the highest rank). We plotted the log of the count in the full corpus against the abbreviation index in red. We also plotted the log of the count in the MC-WSRS fine-tuning dataset against the index, which is a sample of the web-corpus. The sampling methodology results in a high proportion of the low index abbreviations and limits the sampling of medium to high index abbreviations, creating a more balanced dataset.

Supplementary Figure 2: Number of labeled expansions per snippet in finetuning vs synthetic snippets

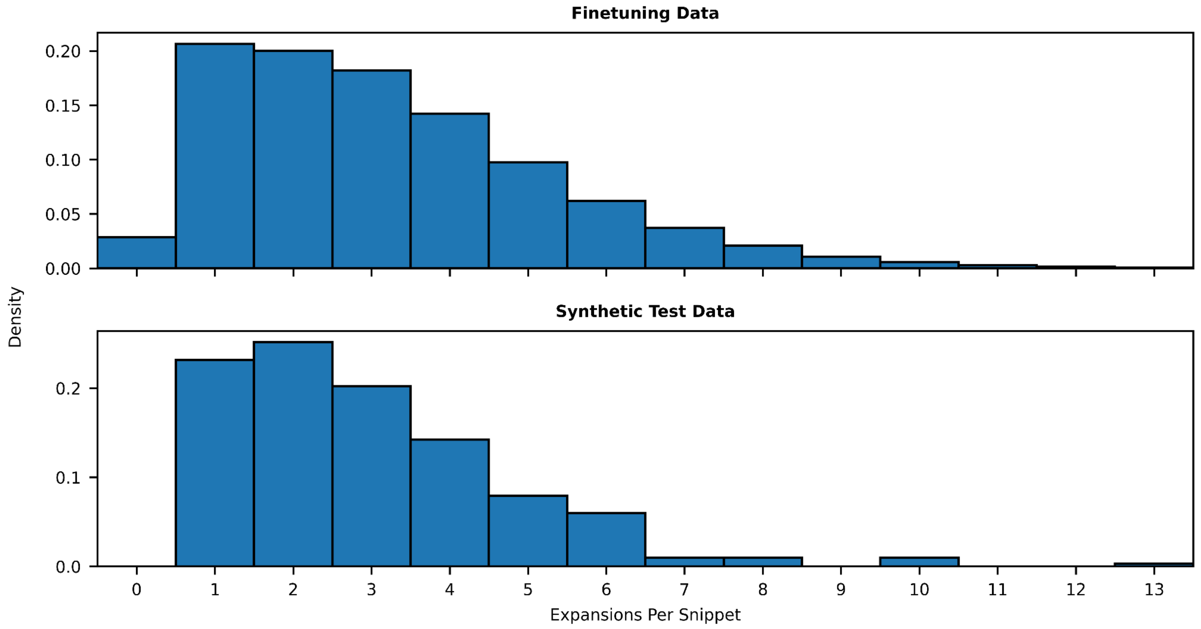

A histogram of the number of abbreviations per snippet in the MC-WSRS validation fine-tuning dataset (a random 5% subset of the entire fine-tuning dataset) (top) compared to the same histogram in the synthetic data (bottom). This roughly similar distribution between the two datasets rules out the possibility of domain shift manifesting through different numbers of abbreviations for simultaneous detection and expansion in a single snippet.

Supplementary Figure 3: Diagram of standard, iterative, and elicitive inference techniques

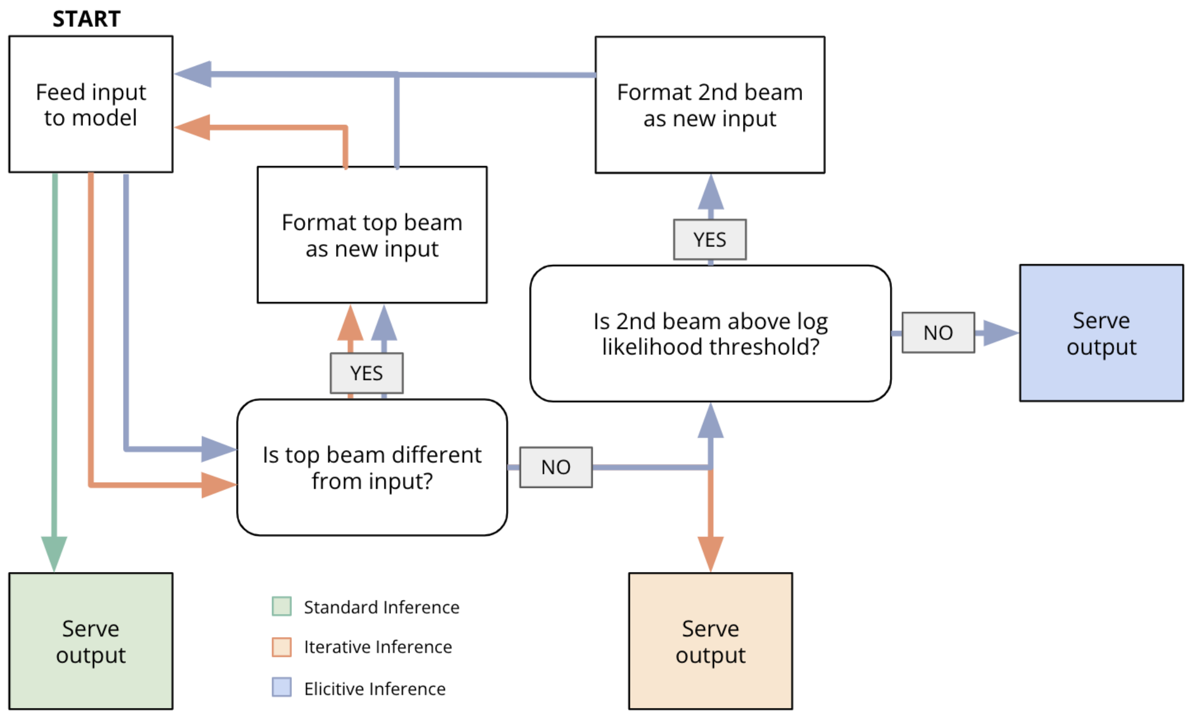

A flowchart illustrating the multiple model inference techniques mentioned in the paper. Each method elicits increasingly aggressive abbreviation detection and expansion in a way that is constrained by the model's posteriors.

Supplementary Table 1: Results on the synthetic dataset for T5 models trained on MC-WSRS + baseline most common expansion

	Inference Type	Detection recall	Detection precision	Expansion accuracy	Total accuracy
T5 60M	Standard	0.897 (0.875, 0.919)	0.989 (0.981, 0.996)	0.931 (0.913, 0.950)	0.835 (0.809, 0.861)
	Iterative	0.964 (0.953, 0.976)	0.990 (0.982, 0.996)	0.929 (0.909, 0.948)	0.896 (0.876, 0.917)
	Elicitive	0.998 (0.994, 1.000)	0.975 (0.964, 0.983)	0.928 (0.911, 0.945)	0.926 (0.906, 0.943)
T5 770M	Standard	0.863 (0.840, 0.887)	0.987 (0.978, 0.995)	0.973 (0.961, 0.984)	0.840 (0.814, 0.865)
	Iterative	0.938 (0.919, 0.955)	0.988 (0.980, 0.995)	0.970 (0.956, 0.982)	0.910 (0.892, 0.930)
	Elicitive	1.000 (1.000, 1.000)	0.948 (0.932, 0.963)	0.963 (0.951, 0.977)	0.963 (0.951, 0.977)
T5 11B	Standard	0.784 (0.751, 0.815)	0.989 (0.982, 0.996)	0.973 (0.959, 0.984)	0.763 (0.727, 0.796)
	Iterative	0.889 (0.863, 0.915)	0.990 (0.982, 0.996)	0.976 (0.965, 0.986)	0.868 (0.843, 0.894)
	Elicitive	0.997 (0.991, 1.000)	0.978 (0.968, 0.987)	0.970 (0.958, 0.980)	0.966 (0.954, 0.978)
T5 80B	Standard	0.819 (0.793, 0.845)	0.996 (0.991, 1.000)	0.979 (0.968, 0.989)	0.802 (0.776, 0.829)
	Iterative	0.901 (0.875, 0.922)	0.996 (0.992, 1.000)	0.981 (0.971, 0.989)	0.885 (0.856, 0.906)
	Elicitive	0.991 (0.984, 0.998)	0.993 (0.987, 0.999)	0.979 (0.969, 0.987)	0.970 (0.959, 0.981)
Most common expansion (English words unexpanded)		0.956 (0.941, 0.971)	0.998 (0.994, 1.000)	0.681 (0.647, 0.712)	0.652 (0.618, 0.684)
Most common expansion (English words expanded)		0.999 (0.996, 1.000)	0.634 (0.607, 0.659)	0.682 (0.650, 0.712)	0.681 (0.649, 0.712)

Supplementary Table 1 shows performance on the synthetic test set by various T5 models fine-tuned on MC-WSRS. The bottom two rows represent baseline methods in which each abbreviation is replaced with the expansion that appears most frequently in the MC web crawl. In the first row, any abbreviations labeled as English words are left unexpanded, and in the second row, they are always expanded. This highlights an unavoidable detection recall-precision tradeoff for such automated systems concerning English word abbreviations. n=400 bootstrap samples of the 302 synthetic snippets, each of which contains a different collection of abbreviations. Point estimate from original sample and 95% confidence intervals calculated using reporting the 2.5 and 97.5 percentile values for each metric

across the samples.

Supplementary Table 2: Results on all clinical notes datasets for T5 80B model (elicitive inference) fine-tuned on MC-WSRS

DR=Detection Recall, EA=Expansion Accuracy, TA=Total Accuracy

	Ambiguous<br>• 241 abbrev<br>• 405 abbrev-expansions<br>• 668 instances			Unambiguous<br>• 143 abbrev<br>• 225 instances			All<br>• 384 abbrev<br>• 548 abbrev-expansions<br>• 893 instances		
Synthetic	DR	EA	TA	DR	EA	TA	DR	EA	TA
	0.990 (0.981, 0.997)	0.971 (0.957, 0.983)	0.961 (0.946, 0.975)	0.996 (0.983, 1.000)	1.000 (1.000, 1.000)	0.996 (0.986, 1.000)	0.991 (0.984, 0.998)	0.979 (0.969, 0.987)	0.970 (0.959, 0.981)
	Ambiguous<br>• 61 abbrev<br>• 119 abbrev-expansions<br>• 20,471 instances			Unambiguous<br>• 3 abbrev<br>• 1,043 instances			All<br>• 64 abbrev<br>• 122 abbrev-expansions<br>• 21,514 instances		
CASI	DR	EA	TA	DR	EA	TA	DR	EA	TA
	0.967 (0.964, 0.969)	0.949 (0.946, 0.952)	0.917 (0.914, 0.921)	0.995 (0.990, 0.999)	0.999 (0.997, 1.000)	0.994 (0.989, 0.998)	0.968 (0.966, 0.971)	0.951 (0.949, 0.954)	0.921 (0.918, 0.925)
	Ambiguous<br>• 872 abbreviations<br>• 2,259 abbreviation-expansions<br>• 10,649 instances			Unambiguous<br>• 1,613 abbreviations<br>• 6,223 instances			All<br>• 2,485 abbreviations<br>• 3,872 abbreviation-expansions<br>• 16,872 instances		
MIMIC-III	DR	EA	TA	DR	EA	TA	DR	EA	TA
Common (N>=500 in MIMIC-III)<br>• 522 abbreviations<br>• 7,367 instances	0.995 (0.993, 0.997)	0.956 (0.952, 0.962)	0.952 (0.947, 0.958)	0.985 (0.978, 0.990)	0.999 (0.998, 1.000)	0.984 (0.978, 0.990)	0.993 (0.991, 0.995)	0.964 (0.960, 0.968)	0.958 (0.953, 0.961)
Uncommon (50<N<500 in MIMIC-III)<br>• 632 abbreviations<br>• 4,817 instances	0.997 (0.995, 0.999)	0.925 (0.917, 0.935)	0.922 (0.914, 0.931)	0.995 (0.992, 0.998)	0.992 (0.987, 0.996)	0.987 (0.981, 0.991)	0.996 (0.994, 0.998)	0.949 (0.943, 0.955)	0.945 (0.939, 0.952)
Rare (0<N<50 in MIMIC-III)<br>• 820 abbreviations<br>• 3,114 instances	0.999 (0.998, 1.000)	0.936 (0.923, 0.949)	0.935 (0.922, 0.949)	0.993 (0.989, 0.997)	0.987 (0.981, 0.991)	0.980 (0.973, 0.986)	0.996 (0.994, 0.998)	0.965 (0.958, 0.972)	0.961 (0.954, 0.968)

All	0.996 (0.995, 0.997)	0.944 (0.940, 0.948)	0.940 (0.936, 0.945)	0.993 (0.991, 0.995)	0.992 (0.989, 0.994)	0.985 (0.982, 0.988)	0.995 (0.994, 0.996)	0.961 (0.959, 0.964)	0.957 (0.953, 0.960)
i2b2-2014	Ambiguous<br>• 609 abbrev<br>• 1,242 abbrev-expansions<br>• 3,422 instances			Unambiguous<br>• 671 abbrev<br>• 1,665 instances			All<br>• 1,280 abbrev<br>• 1,913 abbrev-expansions<br>• 5,087 instances		
	DR	EA	TA	DR	EA	TA	DR	EA	TA
	0.996 (0.994, 0.998)	0.954 (0.947, 0.960)	0.950 (0.944, 0.958)	0.999 (0.998, 1.000)	0.996 (0.992, 0.999)	0.995 (0.991, 0.998)	0.997 (0.996, 0.999)	0.967 (0.963, 0.972)	0.965 (0.960, 0.970)

This table shows a more detailed breakdown of the results on all four external test sets for the T5 80B model fine-tuned on MC-WSRS and combined with elicitive inference. Results are separately reported for abbreviations with and without ambiguity, defined by the presence or absence of more than one expansion in our dictionary, respectively. For the MIMIC-III dataset, we also report results separately for abbreviations based on their frequency in the entire MIMIC-III discharge notes dataset, which we group into three roughly equal-sized categories: common, uncommon, and rare. n=400 bootstrap samples of the snippets, each of which contains a different collection of abbreviations. Point estimate from original sample and 95% confidence intervals calculated using reporting the 2.5 and 97.5 percentile values for each metric across the samples.

Supplementary Table 3: Illustrative human mistakes from human evaluation

Abbreviations and expansions are bolded, with correct expansions highlighted in green and unexpanded or incorrect expansions highlighted in red.

Original string	Human group and output	Model output	Comment
5 **yo m** presenting with multiple burns, encompassing **aprx** 45% **tbsa**.	Layperson without Google<br>5 **years old male** presenting with multiple burns, encompassing **approximate** 45% **tbsa**	5 **year old male** presenting with multiple burns, encompassing **approximately** 45% **total body surface area.**	Lay people without Google could reasonably figure out colloquial terms like "years old" but often did not know how to expand medical terms like "tbsa," which the model excelled at
**t bili** is still markedly elevated and patient will	Layperson without Google<br>**t bili** is still markedly	**total bilirubin** is still markedly elevated and	A common pattern is for lay people not to understand abbreviations

require biliary drainage with **ir** prior to initiation of **chemo**	elevated and patient will require biliary drainage with **infrared red** prior to initiation of **chemotherapy**	patient will require biliary drainage with **interventional radiology** prior to initiation of **chemotherapy**	like "t bili" or to interpret phrases with familiar but incorrect terms ("IR" as "infrared" rather than the correct "interventional radiology")
#hypthyroidism: **t3** low with elevated **tsh** suggestive of primary hypothyroidism. plan to start **trt**	Layperson with Google<br>#hypthyroidism: **triiodothyronine** low with elevated **thyroid stimulating hormone** suggestive of primary hypothyroidism. plan to start **testosterone replacement therapy**	#hypthyroidism: **triiodothyronine** low with elevated **thyroid-stimulating hormone** suggestive of primary hypothyroidism. plan to start **thyroid replacement therapy**	Even with access to Google, knowing how to contextually disambiguate abbreviations like "trt" and "cts" was difficult
started argatroban drip **2/2 hit cb bl civ dvt**	Layperson with Google<br>started argatroban drip **2/2 hit cb bl common iliac vein deep vein thrombosis**<br>Physician<br>started argatroban drip **secondary to heparin-induced thrombocytopenia complicated by bilateral civ deep vein thrombosis**	started argatroban drip **secondary to heparin induced thrombocytopenia complicated by bilateral common iliac vein deep vein thrombosis**	With Google, laypersons were able to understand medical abbreviations like "civ" that not even all attendings understood natively, although common phrasing like "2/2" and "cb" were still hard to parse.
after presenting to the **op** orthopedic clinic, **pt** was instructed to undergo **mri** which demonstrated 2 **cm** soft tissue mass in the **r** ft. most likely benign etiology	Physician<br>after presenting to the **op** orthopedic clinic, **patient** was instructed to undergo **magnetic resonance imaging** which demonstrated 2 **cm** soft tissue mass in the **right** foot. most likely benign etiology	after presenting to the **outpatient** orthopedic clinic, **patient** was instructed to undergo **magnetic resonance imaging** which demonstrated 2 **centimeter** soft tissue mass in the **right** foot. most likely benign etiology	Attending physicians had qualitatively two major error types: not recognizing unfamiliar abbreviations (e.g. "op" and "civ") or not expanding terms that are so commonly not expanded that they are left as abbreviations (e.g. keeping "cm" unchanged rather than expanding to "centimeters"

This table shows illustrative examples of the mistakes made by human translators of various levels of expertise.

Supplementary Table 4: Results on the synthetic dataset for T5 models trained on C4-WSRS

	Inference Type	Detection recall	Detection precision	Expansion accuracy	Total accuracy
T5 60M	Standard	0.807 (0.784, 0.831)	0.986 (0.978, 0.993)	0.914 (0.892, 0.933)	0.738 (0.707, 0.766)
	Iterative	0.908 (0.892, 0.925)	0.988 (0.978, 0.995)	0.911 (0.886, 0.928)	0.828 (0.800, 0.850)
	Elicitive	0.992 (0.985, 0.998)	0.959 (0.947, 0.971)	0.892 (0.870, 0.910)	0.885 (0.864, 0.905)
T5 770M	Standard	0.772 (0.743, 0.798)	0.987 (0.979, 0.994)	0.945 (0.927, 0.960)	0.729 (0.698, 0.757)
	Iterative	0.922 (0.903, 0.936)	0.988 (0.981, 0.994)	0.950 (0.935, 0.965)	0.876 (0.853, 0.898)
	Elicitive	0.998 (0.994, 1.000)	0.981 (0.972, 0.990)	0.944 (0.927, 0.961)	0.942 (0.926, 0.959)
T5 11B	Standard	0.745 (0.714, 0.769)	0.988 (0.981, 0.996)	0.959 (0.945, 0.974)	0.714 (0.684, 0.740)
	Iterative	0.889 (0.869, 0.909)	0.990 (0.984, 0.996)	0.961 (0.948, 0.973)	0.854 (0.832, 0.877)
	Elicitive	0.998 (0.994, 1.000)	0.976 (0.966, 0.986)	0.958 (0.946, 0.972)	0.956 (0.944, 0.970)

Supplementary Table 4 shows performance on the synthetic test set by T5 models fine-tuned on C4-WSRS, which consists of C4 text modified by web-scale reverse substitution. n=400 bootstrap samples of the 302 synthetic snippets, each of which contains a different collection of abbreviations. Point estimate from original sample and 95% confidence intervals calculated using reporting the 2.5 and 97.5 percentile values for each metric across the samples.

Supplementary Table 5: Results on all clinical notes datasets for T5 11B model (elicitive inference) fine-tuned on C4-WSRS

DR=Detection Recall, EA=Expansion Accuracy, TA=Total Accuracy									
Synthetic	Ambiguous<br>• 241 abbrev<br>• 405 abbrev-expansions<br>• 668 instances			Unambiguous<br>• 143 abbrev<br>• 225 instances			All<br>• 384 abbrev<br>• 548 abbrev-expansions<br>• 893 instances		
	DR	EA	TA	DR	EA	TA	DR	EA	TA
	0.997<br>(0.993, 1.000)	0.952<br>(0.937, 0.968)	0.949<br>(0.936, 0.966)	1.000<br>(1.000, 1.000)	0.978<br>(0.957, 0.996)	0.978<br>(0.957, 0.996)	0.998<br>(0.994, 1.000)	0.958<br>(0.944, 0.972)	0.956<br>(0.941, 0.970)
CASI	Ambiguous<br>• 61 abbrev<br>• 119 abbrev-expansions<br>• 20,471 instances			Unambiguous<br>• 3 abbrev<br>• 1,043 instances			All<br>• 64 abbrev<br>• 122 abbrev-expansions<br>• 21,514 instances		
	DR	EA	TA	DR	EA	TA	DR	EA	TA
	0.861<br>(0.856, 0.866)	0.953<br>(0.950, 0.957)	0.821<br>(0.816, 0.827)	0.930<br>(0.913, 0.946)	0.999<br>(0.997, 1.000)	0.929<br>(0.912, 0.943)	0.864<br>(0.860, 0.869)	0.956<br>(0.953, 0.959)	0.826<br>(0.821, 0.832)
MIMIC-III	Ambiguous<br>• 872 abbreviations<br>• 2,259 abbreviation-expansions<br>• 10,649 instances			Unambiguous<br>• 1,613 abbreviations<br>• 6,223 instances			All<br>• 2,485 abbreviations<br>• 3,872 abbreviation-expansions<br>• 16,872 instances		
	DR	EA	TA	DR	EA	TA	DR	EA	TA
Common<br>(N>=500 in MIMIC-III)<br>• 522 abbreviations<br>• 7,367 instances	0.979<br>(0.976, 0.983)	0.938<br>(0.931, 0.944)	0.919<br>(0.911, 0.925)	0.983<br>(0.977, 0.990)	0.997<br>(0.994, 0.999)	0.980<br>(0.974, 0.988)	0.980<br>(0.977, 0.983)	0.949<br>(0.944, 0.954)	0.930<br>(0.925, 0.936)
Uncommon<br>(50<N<500 in MIMIC-III)<br>• 632 abbreviations<br>• 4,817 instances	0.992<br>(0.989, 0.995)	0.906<br>(0.897, 0.917)	0.899<br>(0.889, 0.911)	0.985<br>(0.979, 0.991)	0.995<br>(0.990, 0.998)	0.980<br>(0.972, 0.985)	0.990<br>(0.987, 0.993)	0.938<br>(0.930, 0.944)	0.928<br>(0.920, 0.935)
Rare<br>(0<N<50 in MIMIC-III)<br>• 820 abbreviations<br>• 3,114 instances	0.995<br>(0.991, 0.998)	0.916<br>(0.900, 0.931)	0.911<br>(0.895, 0.925)	0.982<br>(0.976, 0.988)	0.988<br>(0.983, 0.993)	0.971<br>(0.962, 0.978)	0.987<br>(0.984, 0.991)	0.958<br>(0.951, 0.965)	0.946<br>(0.938, 0.954)
All	0.985<br>(0.983, 0.988)	0.924<br>(0.919, 0.929)	0.911<br>(0.905, 0.917)	0.984<br>(0.981, 0.987)	0.988<br>(0.985, 0.990)	0.972<br>(0.968, 0.976)	0.985<br>(0.983, 0.987)	0.948<br>(0.945, 0.951)	0.933<br>(0.930, 0.937)
i2b2-2014	Ambiguous<br>• 609 abbrev<br>• 1,242 abbrev-expansions<br>• 3,422 instances			Unambiguous<br>• 671 abbrev<br>• 1,665 instances			All<br>• 1,280 abbrev<br>• 1,913 abbrev-expansions<br>• 5,087 instances		
	DR	EA	TA	DR	EA	TA	DR	EA	TA
	0.999<br>(0.997, 1.000)	0.931<br>(0.923, 0.939)	0.930<br>(0.921, 0.938)	0.997<br>(0.994, 0.999)	0.984<br>(0.978, 0.990)	0.981<br>(0.975, 0.987)	0.998<br>(0.997, 0.999)	0.948<br>(0.942, 0.954)	0.947<br>(0.940, 0.952)

This table shows results for the T5 11B model + elicitive inference fine-tuned on C4-WSRS. n=400 bootstrap samples of the 302 synthetic snippets, each of which contains a different collection of abbreviations. Point estimate from original sample and 95% confidence intervals calculated using reporting the 2.5 and 97.5 percentile values for each metric across the samples.

Supplementary Figure 4: Number of additional inference rounds on synthetic dataset for iterative and elicitive inference

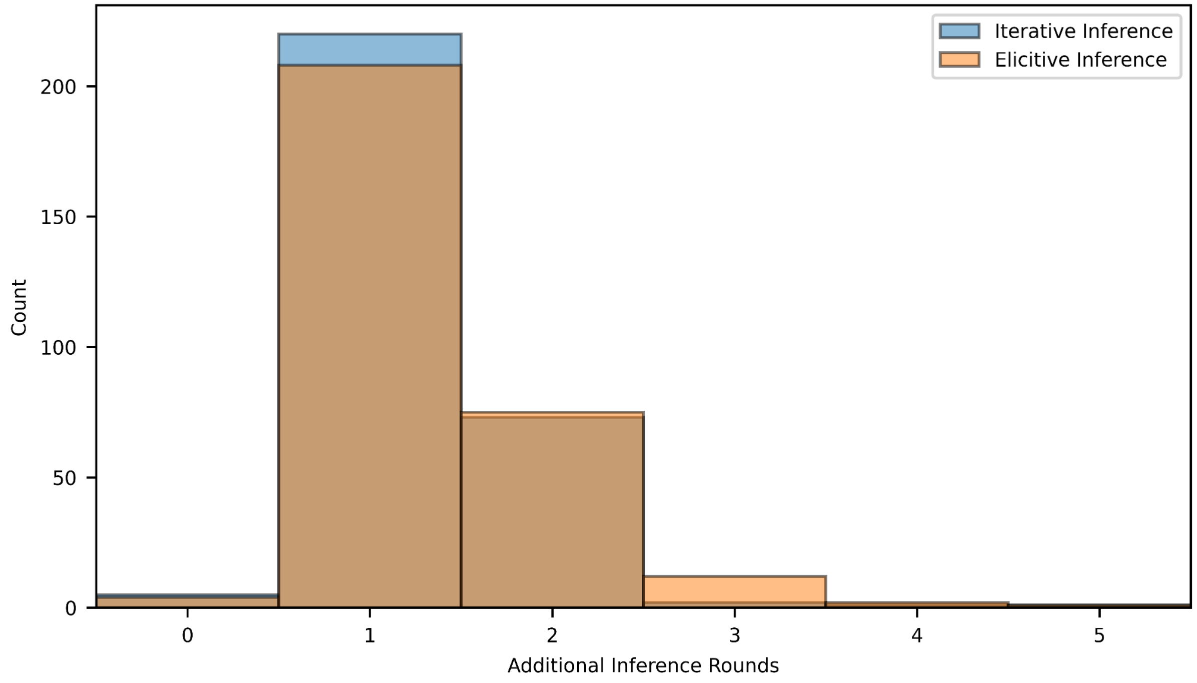

The number of additional rounds of inference conducted for each synthetic snippet before terminating inference for the T5 11B model, for both iterative and elicitive inference. For both techniques, the majority of examples in the synthetic test set required just one additional round of inference. For elicitive inference, more examples required multiple rounds before termination criteria were satisfied.

Supplementary Figure 5: General methodology overview

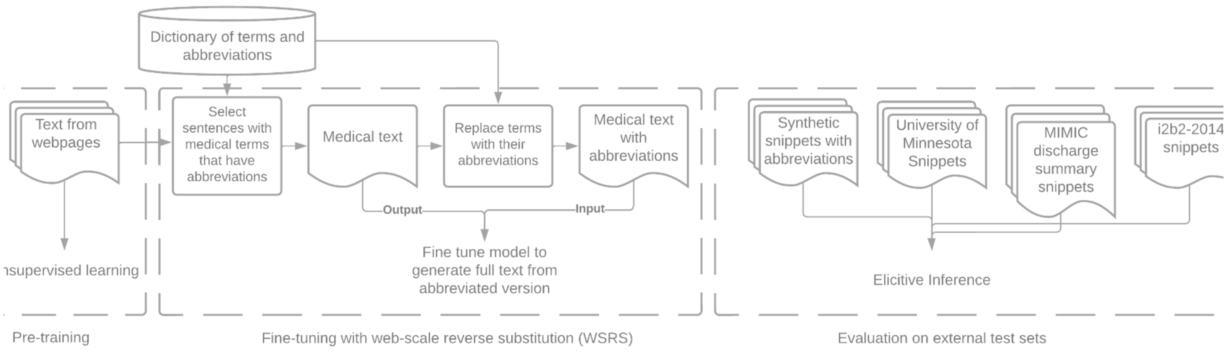

An overview of the entire machine learning system, including the flow of data through the fine-tuning data generation pipeline and the evaluation procedure. A pre-trained langage model (left) was fine-tuned from a web corpus derived from web-scale reverse substitution (middle) and evaluated on four test sets using elicitive inference.

Supplementary Figure 6: Expansion counts in Finetuning data

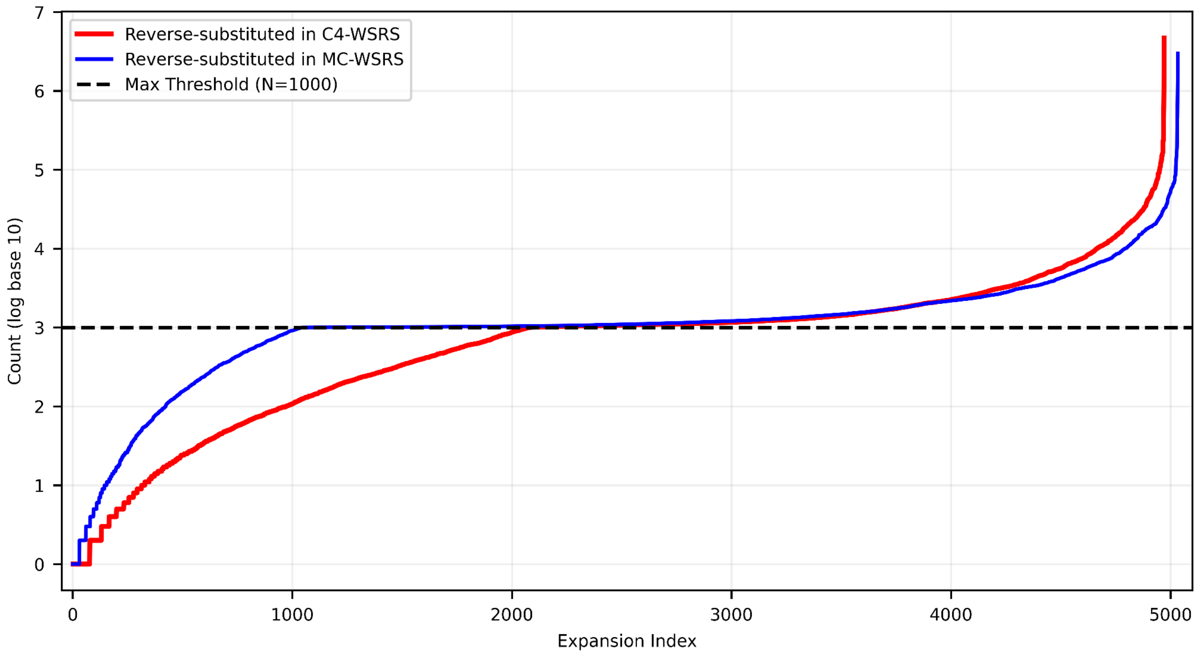

A comparison of the number of reverse substitutions for all expansions in the two finetuning datasets - C4-WSRS and MC-WSRS. The expansion index along the x-axis is derived from ordering expansions by y-value, and the index value for the same expansion is not guaranteed to be the same between the two plotted lines. As discussed in the “Methods” section under “Fine-tuning Dataset Generation with WSRS,” a threshold value of N=1000 was used for both datasets. Compared to the MC web crawl, the C4 web crawl contains fewer examples of the rarer expansions, which may partially explain the observed drop in performance.

Supplementary Algorithm 1: Pseudocode for Web-Scale Reverse Substitution

The pseudocode for the web-scale reverse substitution algorithm. This code is designed to reflect the principles of distributed systems such as Apache Spark. The worker classes can be replicated many times to simultaneously process different partitions of data elements in parallel. Due to differences in libraries and frameworks, the Apache Beam code for generating C4-WSRS, which we release on tensorflow datasets, is slightly different from what is depicted here. Please see the “Methods” section under “Fine-tuning Dataset Generation with WSRS” for more details on those differences.

Algorithm 1 Web-Scale Reverse Substitution

```
Class TextToRSSnippetExamplesWorker()

  # attributes
  map<string, int> abbreviated_count_;

  Method FindRarestMatch(string text)
    string rarest_match;
    int smallest_count = 0;
    for expansion in GetContainedExpansions(text) do
      int current_count = abbreviated_count_[
            (abbreviation, expansion)];
      if (NOT smallest_count
            OR current_count < smallest_count):
      rarest_match = (abbreviation, expansion);
      smallest_count = current_count;
    return rarest_match;
  End Method

  Method AbbreviateText(string text)
    string abbreviated_text;
    for expansion in GetContainedExpansions(text) do
      abbreviated_text += text_before_expansion;
      abbreviation = RandomChoice(valid_abbreviatons[expansion])
      if (RandomUniform(0, 1) < 0.95):
        abbreviated_text += abbreviation;
      else:
        abbreviated_text += expansion;
    abbreviated_text += remaining_text;
    return abbreviated_text;
  End Method
  Method Do(string web_text)
    vector<string> sentences = split(web_text, '. ');
    int sentence_idx = 0;
    while sentence_idx < sentences.size() do
      int num_sentences_sampled = RandomChoice([1, 2, 3]);
      string snippet = StrJoin(
        sentences[sentence_idx: sentence_idx + num_sentences_sampled], \. ") + \.";
      sentence_idx += num_snippets_sampled;
      snippet = ToLowercase(snippet);
      if snippet.length() > 1024: continue;
      tuple<string, string> rarest_match = self.FindRarestMatch(snippet);
      if NOT rarest_match.empty():
        rarest_count = abbreviated_count_[rarest_match];
        should_abbreviate_prob = pow(rarest_count + 1, 1.0);
        if RandomUniform(0, 1) <= should_abbreviate_prob:
          abbreviated_snippet = self.AbbreviateText(snippet);
      else:
        abbreviated_snippet = snippet;
      if abbreviated_snippet.split(' ').size() < 3: continue;
      should_keep_nonabbrev_prob = pow(
        non_abbreviated_examples_num + 1, 1.5);
      if (abbreviated_snippet == snippet
            AND RandomUniform(0, 1) >= should_keep_nonabbrev_prob):
        continue;
      yield (rarest_match, (abbreviated_snippet, snippet));
  End Method
End Class

Class ThresholdExamplesWorker()
  Method Do(string key, vector<tuple<string, string>> examples)
    int remaining_examples = 1000;
    for example in examples do
      yield example;
      remaining_examples--;
      if not remaining_examples: break;
  End Method
End Class

procedure WebScaleReverseSubstitution
  web_texts = ReadData();
  snippet_examples = web_texts.Do(
      TextToRSSnippetExamplesWorker());
  downsampled_snippet_examples = snippet_examples
      .GroupByKey()
      .Do(ThresholdExamplesWorker());
  downsampled_snippet_examples.Save()
```

Supplementary Table 6: Miscellaneous examples

Examples to highlight model capabilities

Domain and Original String	Model Output	Comment
take apap 500mg po q4h **prn** t >101.3 but do not exceed 3gms/d	take acetaminophen 500mg orally every 4 hours **as needed for** temperature >101.3 but do not exceed 3grams/day	"Prn" is expanded into "as needed for"
senna 17.2mg po **prn** if no bm in 2 d	senna 17.2mg orally **as needed** if no bowel movement in 2 days	"Prn" is translated to "as needed" in a way that is grammatically correct
told pt to go to ed if cp, loc, ha, or confusion ow rtc in 2 wks	told patient to go to emergency department if chest pain, loss of consciousness, headache, or confusion otherwise return to clinic in 2 weeks	Return precautions are can be told to patients verbally and documented in the note in unfamiliar terms
45 w w chf with ef 20% s/p aicd and completing wu for lvad	45 woman with congestive heart failure with ejection fraction 20% status post automatic implantable cardiac defibrillator and completing work up for left ventricular assist device	Terminology for advanced cardiac therapies maybe unfamiliar to non-internal medicine physicians
the cv exam has a nr, nr, nl s1 s2, no mrg	the cardiovascular exam has a normal rate, normal rhythm, normal first heart sound and second heart sound, no murmurs rubs or gallops	The model expands "nr" into two separate, correct meanings, which would be difficult to hand-engineer
with ms involvement, sz hx and endo abnormalities, can consider cdgs	with musculoskeletal involvement, seizure history and endocrine abnormalities, can consider carbohydrate deficient glycoprotein syndrome	The model can expand rare diseases
with the underlying copd and pe, there was a double **hit** to his pulm reserve	with the underlying chronic obstructive pulmonary disease and pulmonary embolism, there was a double **hit** to his pulmonary reserve	"hit "for "Heparin induced thromboctyopenia" is a rare disease that can be identified appropriately but can be correctly identified as the word

		"hit" even in similar contexts
please give 4 u aspart if bs > 350	please give 4 units aspart if blood sugar > 350	The abbreviations "u" and "iu" are prohibited by United States regulatory agencies given possible confusion.[22]
heparin comes in 5000 iu/ml and can be administered through an iv route	heparin comes in 5000 international units/milliliter and can be administered through an intravenous route	

This table includes a miscellaneous assortment of input-output examples using the T5 80B model fine-tuned on MC-WSRS and combined with elicitive inference.
