# Supplementary material for: Deciphering clinical abbreviations with a privacy protecting machine learning system
Source: Nat Commun. 2022 Dec 2;13:7456. doi: 10.1038/s41467-022-35007-9 (PMC9718734; doi:10.1038/s41467-022-35007-9)
Supplement: Supplementary file 2 — Reporting Summary [file 41467_2022_35007_MOESM2_ESM.pdf]

nature portfolio

Corresponding author(s): Alvin Rajkomar

Last updated by author(s): 2022-10-8

Reporting Summary

Nature Portfolio wishes to improve the reproducibility of the work that we publish. This form provides structure for consistency and transparency in reporting. For further information on Nature Portfolio policies, see our Editorial Policies and the Editorial Policy Checklist.

Statistics

For all statistical analyses, confirm that the following items are present in the figure legend, table legend, main text, or Methods section.

n/a	Confirmed	
☐	☒	The exact sample size (*n*) for each experimental group/condition, given as a discrete number and unit of measurement
☒	☐	A statement on whether measurements were taken from distinct samples or whether the same sample was measured repeatedly
☒	☐	The statistical test(s) used AND whether they are one- or two-sided<br>*Only common tests should be described solely by name; describe more complex techniques in the Methods section.*
☒	☐	A description of all covariates tested
☒	☐	A description of any assumptions or corrections, such as tests of normality and adjustment for multiple comparisons
☐	☒	A full description of the statistical parameters including central tendency (e.g. means) or other basic estimates (e.g. regression coefficient) AND variation (e.g. standard deviation) or associated estimates of uncertainty (e.g. confidence intervals)
☒	☐	For null hypothesis testing, the test statistic (e.g. *F*, *t*, *r*) with confidence intervals, effect sizes, degrees of freedom and *P* value noted<br>*Give P values as exact values whenever suitable.*
☒	☐	For Bayesian analysis, information on the choice of priors and Markov chain Monte Carlo settings
☒	☐	For hierarchical and complex designs, identification of the appropriate level for tests and full reporting of outcomes
☒	☐	Estimates of effect sizes (e.g. Cohen's *d*, Pearson's *r*), indicating how they were calculated

Our web collection on statistics for biologists contains articles on many of the points above.

Software and code

Policy information about availability of computer code

Data collection	Data for fine-tuning was from snippets of text collected from the public web that were processed with reverse substitution. We have released the code that allows the collection and processing of these snippets to be generated using the publicly available C4 dataset; this is described in more detail in the "Applying WSRS to C4" section of the paper, and the code is available on Tensorflow Datasets (https://www.tensorflow.org/datasets/catalog/c4_wsrs). The manuscript also uses a version of an internal web-crawl and a version of web-scale reverse substitution that relies on proprietary frameworks that cannot be released; the processing is described in detail as pseudocode in Supplementary Algorithm 1. Please refer to the above section “Applying WSRS to C4” for more details on how this implementation is different and what results we were able to achieve on this dataset.
Data analysis	Our training procedure can be exactly replicated using a publicly accessible model training framework such as github.com/google-research/t5x (please refer to the “Model Description” section above for detailed model training hyperparameters). To evaluate trained models, we make a collection of python libraries publicly available at github.com/google-research/google-research/tree/master/deciphering_clinical_abbreviations. The “tokenizer” library contains a custom tokenizer designed for the project, such that abbreviations and expansions are kept as atomic tokens for alignment. The “text_alignment” library contains code to align raw inputs and expanded outputs, as described above in the “Evaluation Metrics” section. The “expansion_attribution” library contains code for converting these alignments into the resulting pairs of abbreviations and their expansions, which can then be used for metric computation in the “evaluation” library. For more detailed usage instructions, refer to the codebase’s README.md file. This repository also contains a script that can be used to reproduce the evaluation of the T5 80B model outputs. The script downloads the relevant files from gs://gresearch/deciphering_clinical_abbreviations and prints the metrics values to stdout. This code is released under the Apache License 2.0.

For manuscripts utilizing custom algorithms or software that are central to the research but not yet described in published literature, software must be made available to editors and reviewers. We strongly encourage code deposition in a community repository (e.g. GitHub). See the Nature Portfolio guidelines for submitting code & software for further information.

Data

Policy information about availability of data

All manuscripts must include a data availability statement. This statement should provide the following information, where applicable:

- Accession codes, unique identifiers, or web links for publicly available datasets
- A description of any restrictions on data availability
- For clinical datasets or third party data, please ensure that the statement adheres to our policy

The overall framework of the available data includes: model parameters of various T5 language models, a version of a public web-crawl (C4) and code to generate fine-tuning examples for the language models, the dictionary used for abbreviations, the synthetic snippets for evaluation, and the publicly available de-identified notes also used for evaluation. The T5 small, T5 large, and T5 11B pre-trained model checkpoints used in this study are publicly available at github.com/google-research/text-to-text-transfer-transformer, and the code to download the C4 corpus used for pre-training these checkpoints is also available in TensorFlow Datasets (https://www.tensorflow.org/datasets/catalog/c4). The 80B model checkpoint, which represents a significant increase in both size and pre-training, is not currently available. The code to download a modified version of C4 which we used for public dataset fine-tuning is also available in Tensorflow Datasets (https://www.tensorflow.org/datasets/catalog/c4_wsrs), but before this dataset can be generated, C4 must be downloaded first so that it can be used as the input dataset. The larger-scale internal web crawl has undergone similar filtering as described by Du et al.48 and is not currently available for download (but the underlying data is accessible through any internet browser). The manually curated abbreviation-expansion dictionary used for this project is available for download at gs://gresearch/deciphering_clinical_abbreviations. Of the four test datasets used for evaluation in this paper, three are publically available from the following sources: MIMIC III (https://physionet.org/content/mimiciii/1.4/). CASI (https://conservancy.umn.edu/handle/11299/137703). i2b2 (https://www.i2b2.org/NLP/DataSets/Main.php). The fourth dataset, which is composed of synthetic snippets covering a range of abbreviations written by clinicians for this project, is also available for download at gs://gresearch/deciphering_clinical_abbreviations. We also make two additional resources available at gs://gresearch/deciphering_clinical_abbreviations: the list of 180 words we manually identified as both abbreviations and English words, and a table of all expansions manually labeled as being clinically equivalent for a given abbreviation (e.g. For the abbreviation “abd,” the expansions “abdomen exam” and “abdominal exam” are considered clinically equivalent). These equivalencies are used to identify model outputs which are not exact matches with, but are clinically equivalent to, the labeled expansions.

Human research participants

Policy information about studies involving human research participants and Sex and Gender in Research.

Reporting on sex and gender	N/A
Population characteristics	N/A
Recruitment	N/A
Ethics oversight	N/A

Note that full information on the approval of the study protocol must also be provided in the manuscript.

Field-specific reporting

Please select the one below that is the best fit for your research. If you are not sure, read the appropriate sections before making your selection.

☒ Life sciences ☐ Behavioural & social sciences ☐ Ecological, evolutionary & environmental sciences

For a reference copy of the document with all sections, see nature.com/documents/nr-reporting-summary-flat.pdf

Life sciences study design

All studies must disclose on these points even when the disclosure is negative.

Sample size

Sample sizes for the test sets can be measured in a variety of ways - unique abbreviation-expansion counts, abbreviation-expansion counts in snippets, and snippet counts. The unique abbreviation-expansion count was selected to include all abbreviations encountered in clinical text (as opposed to general biomedical text in scientific papers) that were mentioned in the literature.

For the synthetic dataset size, we obtained as many examples as we could given the time-availability of the medical specialists who wrote them with the constraint that each snippet had to have at least one unique abbreviation-expansion pair that was provided from the dictionary and sampled such that half were ambiguous.

For the CASI dataset, we used all available examples of abbreviations that were contained in the dictionary (in addition to a few other minor exclusions that are documented in the methods).

For MIMIC-III and i2b2, we used all available data to sample all abbreviation-expansion pairs in our dictionary but we used a procedure to generate balanced and diverse test sets and prevent both common abbreviations and common expansions from dominating the set. The exact

	procedure to achieve the balanced sample is described in the methods section, but we ensured that each abbreviation-expansion was represented at least three times in the test sets. The resulting sample is sufficient for comparison to the literature as it is larger or comparable to the next largest study (Skreta et al).<br><br>For the sample size of how many examples to use for fine-tuning, we used a standard practice of early stopping (stopped training when the loss of the validation set of randomly held out examples stopped decreasing).
Data exclusions	Because our study was intended to assess performance of expanding abbreviations, we excluded snippets from the evaluation set that did not include abbreviations in our dictionary as a pre-established requirement and the small number of snippets with poor quality (which is described in the methods section).
Replication	As part of standard practice, we iterated model development on training and development data and did not use test set/evaluation data until a final model was created. Once that model was created after the fine-tuning process, we applied inference of that static model at least 3 times to each test set and obtained identical results each time. These are the results that are published.<br><br>After this result was obtained, we also re-performed web-scale reverse substitution (a non-deterministic process) process and re-performed experiments twice and obtained similar results.
Randomization	Samples were randomly sorted into train/validation/test sets where applicable
Blinding	Investigators has no insight into the randomization algorithms to split the data and could be considered blinded.

Reporting for specific materials, systems and methods

We require information from authors about some types of materials, experimental systems and methods used in many studies. Here, indicate whether each material, system or method listed is relevant to your study. If you are not sure if a list item applies to your research, read the appropriate section before selecting a response.

Materials & experimental systems

n/a	Involved in the study
☒	☐ Antibodies
☒	☐ Eukaryotic cell lines
☒	☐ Palaeontology and archaeology
☒	☐ Animals and other organisms
☒	☐ Clinical data
☒	☐ Dual use research of concern

Methods

n/a	Involved in the study
☒	☐ ChIP-seq
☒	☐ Flow cytometry
☒	☐ MRI-based neuroimaging
